# Creation of an optic nerve sheath diameter ultrasound model for NeuroICU education

**DOI:** 10.1186/s40779-020-00274-4

**Published:** 2020-09-21

**Authors:** Heidi M. Felix, Kristin A. Rosenbush, Amy M. Lannen, Robert A. Pooley, Jason L. Siegel, Benjamin L. Brown, Melissa L. McMullan, Christina I. Collins, William D. Freeman

**Affiliations:** 1 J. Wayne and Delores Barr Weaver Simulation Center, Mayo Clinic, 4500 San Pablo Road, Jacksonville, FL 32224 USA; 2 Division of Medical Physics, Mayo Clinic, Jacksonville, FL 32224 USA; 3 Department of Critical Care Medicine, Mayo Clinic, Jacksonville, FL 32224 USA; 4 Department of Neurology, Mayo Clinic, Jacksonville, FL 32224 USA; 5 Department of Neurologic Surgery, Mayo Clinic, Jacksonville, FL 32224 USA; 6 Department of Radiology, Mayo Clinic, Jacksonville, FL 32224 USA; 7 Department of Nursing, Mayo Clinic, Jacksonville, FL 32224 USA

**Keywords:** Intracranial pressure, Simulation, Task trainer, Ultrasound

## Abstract

**Background:**

Using ultrasound to measure optic nerve sheath diameter (ONSD) is an emerging bedside technique to noninvasively assess intracranial pressure (ICP) in patients with brain injury. This technique is unique among bedside ultrasonography and is often performed by providers who have no formal ultrasound training. We sought to create a low-cost, 3D, reusable ONSD model to train neurology, neurosurgery, and critical care providers in measuring ICP.

**Results:**

We identified 253 articles, of which 15 were associated with models and 2 with simulation. One gelatin model was reported, upon which we based our initial design. We could not validate the visual findings of this model; however, after constructing multiple beta models, the design most representative of human eye anatomy was a globe made of ballistics gel and either a 3 mm, 5 mm, or 7 mm × 50 mm 3D-printed optic nerve inserted into a platform composed of ballistics gel, all of which sat inside a 3D-printed skull. This model was used to teach ONSD measurements with ultrasound at a continuing medical education event prior to training on a live human model.

**Conclusion:**

A simple 3D ballistic ONSD model allows learners to practice proper hand placement and pressure, basic landmarks, and ONSD measurement prior to operating on a human eye. This model is replicable and sustainable given that the globe and platform are composed of ballistics gel.

## Background

Measuring optic nerve sheath diameter (ONSD) with point-of-care ultrasound is an emerging technique to noninvasively assess intracranial pressure (ICP) in patients with brain injury. Multiple studies have demonstrated that ONSDs between 4.5 mm and 5.8 mm correspond to an ICP greater than 20 mmHg by the criterion-standard external ventricular drain [1–9]. Moreover, Hassen et al. [10] found a similar correlation between computed tomography measurements of ONSDs and ultrasound. As with many procedures, creating a simulation or task trainer is preferred before applying the modality on a patient, and standardized techniques and education need to be implemented for the ultrasound findings to be reliable [8, 11]. Our objectives were to produce an ONSD model that was anatomically accurate when viewed by ultrasound, to implement a task trainer for measuring ONSD and to evaluate the ease of use for implementing this model in the training of neurology, neurosurgery, and critical care advanced practice providers and physicians.

## Results

Our literature search revealed 253 articles. When the search terms were combined with “model,” 15 articles remained. Of these, 2 were porcine models, 2 were simulations, and 1 was a gelatin-based model [12]. We attempted to replicate the gelatin-based model but were unable to reproduce the visual results, so we decided to create our own 3D model. The remaining publications on ONSD appeared to be related to human or other ONSD measurement types, including computed tomography.

### Constructing the ballistics gel eyeball model for ONSD

Ballistics gel, commonly used by police and government agencies in bullet trajectory testing, is a useful tool in simulation. For our purposes, we used a 3-step process to create an eyeball out of the gel. Before embarking on our own mold creation process, commercially available molds were explored, but these products were too large, had seams that showed on ultrasound, or were too difficult to fill with the gel.

A 3-cm plaster eyeball was molded to facilitate creating a hard positive from which we could create a flexible mold. To prepare the plaster eyeball for the molding process, it was sprayed with an acrylic sealer, followed by a mold release agent.

**Fig. 1:**
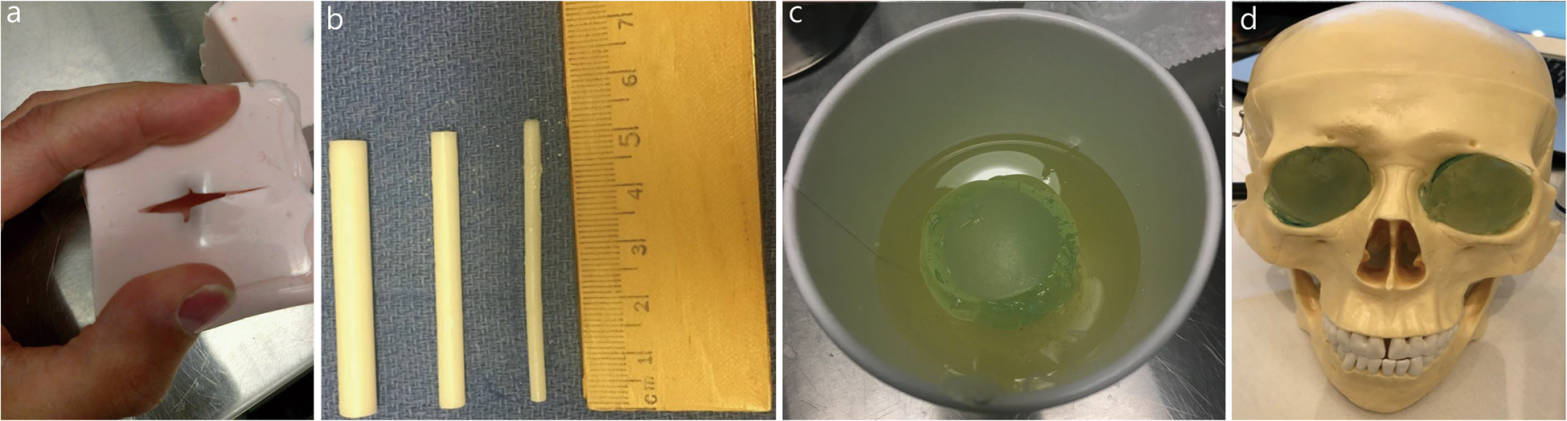
Components of the Model. **a**. Silicone mold. **b**. Three-dimensional printed optic nerves. **c**. Initial eyeball model. **d**. Full head model

To create our mold, we used a small, square, plastic container that we separated into sections by hot gluing a piece of scrap plastic in the center and on the sides to create 4 quadrants. In the future, it may be easier to use small paper cups in place of this plastic container. We mixed Mold Max 20 (Smooth-On, Inc.) according to the package instructions and poured it into 3 of the 4 quadrants. We then placed a plaster ball in each quadrant, ensuring that the ball did not float to the top of the Mold Max 20. Once the Mold Max 20 was cured, we removed it from the plastic container and made a small incision on top of each of the 3 completed pieces (Fig. 1a), through which the balls were removed.

Finally, we melted the ballistics gel and poured it into our molds. It was important to allow ballistics gel to heat completely, and all bubbles were removed when the gel reached peak temperature. This was achieved by heating the gel in a slow cooker until the block of the gel was liquefied (approximately 4 h). When the product was fully melted and free of bubbles, a metal pitcher was carefully dipped into the hot gel, and the gel was quickly and smoothly poured into the molds. Once the gel in the molds cooled, it was removed.

### Constructing the optic nerve

Optic nerves were created from a 3D printer. Multiple lengths were printed in an attempt to create the most realistic ultrasound findings. We printed 1 normal optic nerve measuring 3 mm × 50 mm to simulate normal ICP and 1 abnormal optic nerve measuring 7 mm × 50 mm to simulate elevated ICP (ONSD > 0.5 cm, Fig. 1b). Figure 1c shows the fully constructed initial model, composed of a ballistics gel base with a hollow core in which the optic nerve sat. While we have not yet been able to create the trabeculae noted in Fig. 1, the optic nerve image created allows the user to practice measuring the diameter while simultaneously placing his/her hand and ultrasound probe in a manner that does not place too much pressure on the globe.

### 3D-printed head and eyeball model

**Fig. 2:**
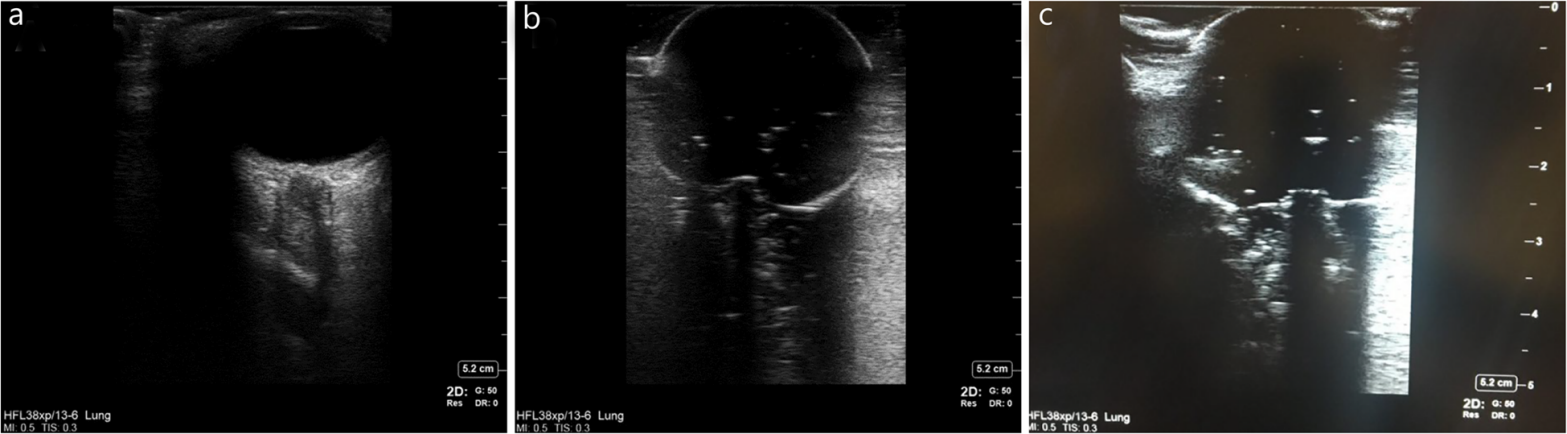
Optic nerve sheath diameter. **a**. Normal human optic nerve sheath diameter. **b**. Optic nerve sheath diameter 3-mm model represents a normal intracranial pressure. **c**. Optic nerve sheath diameter 7-mm model represents an elevated intracranial pressure

We printed a 3D skull model using an existing deidentified head computed tomography from a human patient (Fig. 1d). The skull’s eye sockets allowed for implantation of the removable ballistics gel eyeball models. Ultrasound findings of a normal human ONSD versus our modeled 3-mm and 7-mm ONSDs are shown in Fig. 2a, b, and c, respectively. SonoSite X-Porte ultrasound equipment (FUJIFILM SonoSite, Inc., Bothell, WA, US) was used to obtain the images. US Food and Drug Administration power limits are low for orbits, and only systems approved for orbital imaging should be used.

This model is used widely in training at various conferences sponsored by our institution for a variety of health care providers. Participants continually rate the training model effective in its objective of teaching hand placement for obtaining images, measuring images, and learning how much pressure to place on an orbit while obtaining images.

## Discussion

Point-of-care ultrasound is an invaluable tool for clinical diagnosis, and due to Moore’s law, the cost, size, and speed of computing are becoming smaller, faster, and less expensive. Ultrasound has global health implications given its wide safety index in proper clinical contexts. Icahn School of Medicine at Mount Sinai, for example, provided pocket ultrasounds to their medical students to train their future physicians in ultrasound in addition to their other physical examination skills, such as stethoscopes for auscultation. Rajajee et al. [7] demonstrated receiver operating characteristics of ONSD against the criterion-standard external ventricular drain. Their study showed that when measured 0.3 cm from the optic nerve head, an ONSD of 0.5 cm or greater correlated with an ICP greater than 20 mmHg, a sensitivity of 96% (95% CI 91–99%), and a specificity of 94% (95% CI 92–96%) [7].

Limitations of the study include the lack of a known standardized model to train physicians and other health care providers in ultrasound ONSD measurement. Using the available literature, we attempted to recreate a gelatin-based ONSD model [12]; we believe this model will improve on the noninvasive ICP measurement training that currently exists. We recognize that this is only a prototype, but we are encouraged by its initial results. Future directions include creating a simulated eyelid to overlie the model, creating more optic nerves to represent a variety of clinical scenarios (especially borderline measurements), amending the optic disc to be more elevated in the larger optic nerve model, and creating a material to represent trabeculae to further enhance anatomical accuracy [13]. We found that there was an education gap at our own medical center in terms of basic knowledge between reading an article and the actual hands-on skills needed to acquire ONSD at different levels (cup model, 3D-printed head model, live human volunteer or patient). We also found our cup and 3D-printed model to be educationally valuable among neurologists, intensivists, nurse practitioners, and physician assistants. We also measured educational feedback at a CME course using all three levels of models (cup, 3D-printed head, and live human volunteer or patient), which was rated as excellent in all workshop participants.

The ability to confidently measure ONSD stands to augment the care of patients diagnosed with subarachnoid hemorrhage, subdural hematoma, intraventricular hemorrhage, traumatic brain injury, and cerebral edema from acute liver failure. Risks of bedside ultrasound are minimal. However, if the operator is not cognizant of probe position, pressure, and technique, ultrasound of the orbit can cause discomfort, nausea, vomiting, vagal response, or even injury to the globe [7]. The ability to enhance motor skills for this procedure while gaining the skills to measure the optic nerve is an important component of this task trainer. Our task trainer incorporates both a normal-sized optic nerve and an optic nerve representative of increased ICP, so the learner can have the benefit of distinguishing both. Furthermore, the trainer can be placed into an anatomically correct skull model to further enhance hand and probe placement. Last, because this model uses ballistics gel, it is sustainable and does not require special storage.

In summary, we report a progressive, engineered ONSD model for human noninvasive ICP simulation education based on the available literature and structured iteration and feedback. We believe that ONSD measurement is a critical tool for neurologists, neurointensivists, medical intensivists, and other health care providers globally to triage patients at risk for elevated ICP. We believe this technology can be used anywhere ultrasound can be provided (e.g., emergency departments, intensive care units), as well as in underserved regions of the globe and even in the battlefield for patients with traumatic brain injury.

## Conclusions

We describe a practical medical simulation in 3 phases for training multidisciplinary teams in ultrasound ONSD measurement. This model is replicable at a relatively low cost and can be taught at other medical centers and CME events. Additionally, given the noninvasive aspects of ultrasound ONSD measurement, we find these methods useful for training learners of different medical levels of expertise before trying this measurement directly on patients in the intensive care setting.

## Methods

This study did not include research involving humans, so informed consent and institutional review board approval were not required.

We searched the National Library of Medicine for articles relevant to measuring ONSD by ultrasonography, using the search terms “optic nerve sheath diameter” and “ultrasonography.” Concurrently, we reviewed available materials on the market for modeling in simulation and studied normal ophthalmic and intracranial anatomy to inform our process.

We divided the project into 3 phases. Phase I was a simplistic *cup and ballistics gel eyeball* model. In this phase, we investigated the ultrasound properties of ONSD appearance similar to human ONSD measurement. We used several small fixed diameters, or *discs*, replicating different ONSDs, followed by ONSD measurement of the discs. This was done to visualize the readiness level of the model before building a three-dimensional (3D) printed skull. Two investigators (WDF and JLS) who were experienced in measuring ONSDs in human patients performed several measurements in the simulation center.

In Phase II, we built a 3D-printed skull and embedded the ballistics gel models described in Phase I. This design allowed the eyeball to be interchangeable and provided a better experience for learners to position their hand and practice ultrasound techniques, such as fanning, tilting, and rotating the ultrasound probe.

In Phase III, we took the 3D-printed head model to a live continuing medical education (CME) course. The instructors (WDF and JLS) presented the Phase I and II models for the participants to practice hand placement, probe manipulation, and anatomy identification. They then performed ONSD measurements on conference staff who volunteered as live models.

## Data Availability

The dataset supporting the conclusions of this article is included in the article.

## Abbreviations

3D: Three-dimensional
CME: Continuing medical education
ICP: Intracranial pressure
ONSD: Optic nerve sheath diameter
